# Quadruple-Cation Wide-Bandgap Perovskite Solar Cells with Enhanced Thermal Stability Enabled by Vacuum Deposition

**DOI:** 10.1021/acsenergylett.2c00304

**Published:** 2022-03-18

**Authors:** Isidora Susic, Lidón Gil-Escrig, Francisco Palazon, Michele Sessolo, Henk J. Bolink

**Affiliations:** Instituto de Ciencia Molecular, Universidad de Valencia, C/Catedrático J. Beltrán 2, 46980 Paterna, Spain

## Abstract

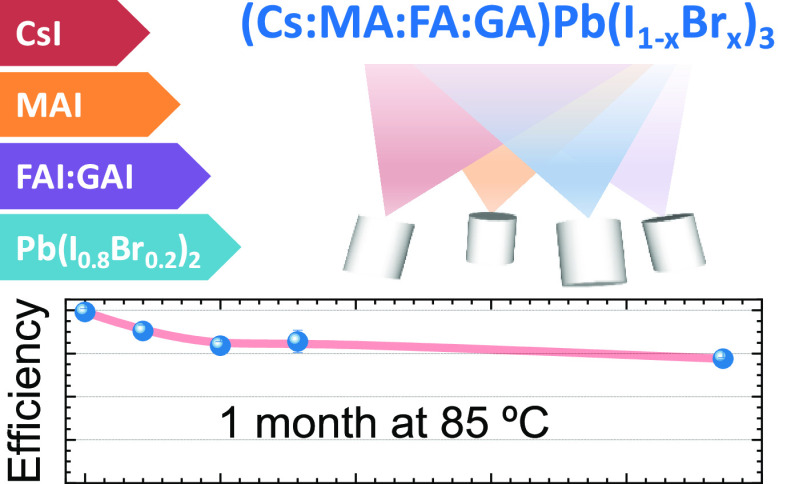

Vacuum processing of multicomponent perovskites is not straightforward, because the number of precursors is in principle limited by the number of available thermal sources. Herein, we present a process which allows increasing the complexity of the formulation of vacuum-deposited lead halide perovskite films by multisource deposition and premixing both inorganic and organic components. We apply it to the preparation of wide-bandgap CsMAFA triple-cation perovskite solar cells, which are found to be efficient but not thermally stable. With the aim of stabilizing the perovskite phase, we add guanidinium (GA^+^) to the material formulation and obtained CsMAFAGA quadruple-cation perovskite films with enhanced thermal stability, as observed by X-ray diffraction and rationalized by microstructural analysis. The corresponding solar cells showed similar performance with improved thermal stability. This work paves the way toward the vacuum processing of complex perovskite formulations, with important implications not only for photovoltaics but also for other fields of application.

Among emerging photovoltaic (PV) technologies, thin-film solar cells based on organic–inorganic (hybrid) lead halide perovskites (herein called perovskites) are the most widely investigated. High-quality semiconducting perovskites can be prepared with simple and potentially inexpensive processes^1−6^ because of their high tolerance to defects,^7,8^ low trap density, and long carrier diffusion length.^9−14^ As a result, the efficiency of single-junction perovskite cells has rapidly grown,^15^ reaching a power conversion efficiency (PCE) approaching 26%.^16^ An important feature of perovskites is the possibility to fine-tune their bandgap by compositional engineering,^17−20^ making them suitable for single- and multi-junction solar cells.^21−27^ In tandem devices, perovskite compositions with wide bandgaps (>1.65 eV) are needed in order to exceed the theoretical efficiency limit of single-junction solar cells.^28,29^ These compositions are obtained by using mixed iodide/bromide formulations, where mixed A-site cations are typically employed to improve the photo- and thermal stability of the compounds.^30−36^ The vast majority of reports on (wide-bandgap) perovskite solar cells are based on solution-processing techniques. Vacuum co-sublimation is less explored, although its superior control over the film thickness and composition and its intrinsic solvent-free nature are of special relevance for the fabrication of complex multilayer architectures.^37−47^ Vacuum-deposited MAPb(I_1–*x*_Br_*x*_)_3_ films and solar cells have been prepared with two- and three-source processes, where stable films can be obtained only with *x* up to 0.2 (1.7 eV).^48,49^ For higher bromide content, the perovskite demixes into iodide- and bromide-rich phases in a process known as “halide segregation”,^30,50,51^ which can be alleviated by adding mixed A-site cations such as cesium and formamidinium (Cs^+^, FA^+^).^30−32^ Mixed-cation and mixed-halide wide-bandgap perovskites of the type FA_1–*n*_Cs_*n*_Pb(I_1–*x*_Br_*x*_)_3_ have been also prepared via vacuum deposition, either with a three-source process using PbI_2_, CsBr, and formamidinium iodide (FAI),^52,53^ or with four sources using FAI, CsI, PbI_2_, and PbBr_2_ as the precursors.^54^ The latter method, relying on the simultaneous sublimation of the two lead halides, allows to decouple and control the relative bromide/cesium content. We showed that wide-bandgap Cs_0.5_FA_0.4_MA_0.1_Pb(I_0.83_Br_0.17_)_3_ perovskite films can be prepared in a four-source cosublimation process, from PbI_2_, CsBr, formamidinium iodide (FAI), and methylammonium iodide (MAI) precursors, where CsBr was used simultaneously as the source of Cs^+^ and Br^–^.^55^ However, in order to increase the bandgap (*E*_g_ > 1.7 eV), a substantial amount of Br^–^ has to be incorporated, resulting in an equally large cesium concentration. The excess cesium was found to cause an irregular morphology, leading to poor device performance.^55^ Recently, we demonstrated the possibility to sublime mixed-metal halide precursors from a single source, by prealloying two precursors via melting them in nitrogen atmosphere at ambient pressure.^56^ This strategy liberates one thermal source which can be used to add another component in the perovskite deposition process.

In this work we demonstrate the vacuum processing of triple-cation CsMAFA perovskite films from four sources, subliming simultaneously CsI, MAI, FAI, and a prealloyed mixture of PbI_2_ and PbBr_2_. This process leads to homogeneous perovskite films and efficient wide-bandgap perovskite solar cells. However, the perovskite films and devices were found to be thermally unstable upon stressing at 85 °C. Hence, with the aim of stabilizing the structure of the CsMAFA perovskite, we added a fourth A-site cation. Guanidinium (GA^+^) has been reported to stabilize both FA- and MA-based perovskites because of an increased number of H bonds with favorable orientation within the inorganic framework.^57−62^ In addition, GA^+^ can be incorporated (to a certain extent) in a lead halide perovskite lattice without breaking the 3D structure, as its ionic radius (278 pm) is only slightly larger as compared to FA^+^ (253 pm).^59^ To add yet another component, we took advantage of the similar sublimation properties of GAI and FAI and sublimed them together from a single thermal source. This led to a four-source deposition process with six precursors, namely, CsI, MAI, FAI, GAI, PbI_2_, and PbBr_2_, with FAI/GAI and PbI_2_/PbBr_2_ sublimed from single sources. The as-prepared films were found to be highly stable upon thermal stress and light soaking, and the corresponding solar cells show PCE similar to that of the triple-cation counterpart. Importantly, the thermal stability of the perovskite composition translates to solar cells with long lifetime at 85 °C, which is comparable to best-in-class pure iodide perovskites prepared by vacuum deposition.

Triple-cation perovskite films were prepared from four sources, subliming simultaneously CsI, MAI, FAI, and a prealloyed mixture of PbI_2_ and PbBr_2_ (schematics in Figure 1a; details in the Supporting Information). As-prepared films were analyzed by scanning electron microscopy (SEM, Figure S1), showing randomly oriented grains with typical size in the 100 nm range, as frequently observed for vacuum-deposited perovskites. The X-ray diffraction (XRD) characterization of the as-deposited triple-cation perovskite thin film is shown in Figure S2. The signal can be fitted considering a single cubic perovskite phase (space group *Pm*3̅*m*; see the Supporting Information for further details) with a lattice parameter of 6.22 Å, with only a small contribution of PbI_2_ as well as the underlying ITO substrate. The lattice parameter is smaller than that of pure-iodide hybrid organic–inorganic cubic perovskites such as FA_1–*x*_MA_*x*_PbI_3_ because of the incorporation of the smaller anion Br^–^ and cation Cs^+^.^63^ Moreover, the film shows a slight preferential crystalline orientation perpendicular to the (100) plane, though reflections for other directions are not completely suppressed. The absorbance spectra of a 500 nm thick wide-bandgap CsMAFA perovskite film is reported in Figure S3a, showing the expected perovskite absorption profile, with absorbance >1 for wavelengths below approximately 550 nm and *E*_g_ = 1.73 eV, as estimated from the corresponding Tauc plot (Figure S3b). The photoluminescence spectrum (Figure S3c), obtained upon illumination with a 515 nm laser at carrier concentration equal to 1 sun illumination, shows a maximum at 1.735 eV and no halide segregation in as-prepared films. In view of the promising characteristics of the triple-cation CsMAFA wide-bandgap perovskite, we have used them to prepare thin-film solar cells in the p-i-n configuration (details of the device preparation are reported in the Supporting Information). Briefly, patterned indium tin oxide (ITO) transparent electrodes were coated with a thin layer (∼5 nm) of poly(triarylamine) (PTAA) as the hole transport layer (HTL). Afterward, a 500 nm thick perovskite film was deposited on top and capped with an electron transport layer (ETL, C_60_, 25 nm). A thin (8 nm) film of bathocuproine (BCP) was used to ensure ohmic contact between the ETL and a silver electrode (100 nm thick).

**Figure 1:**
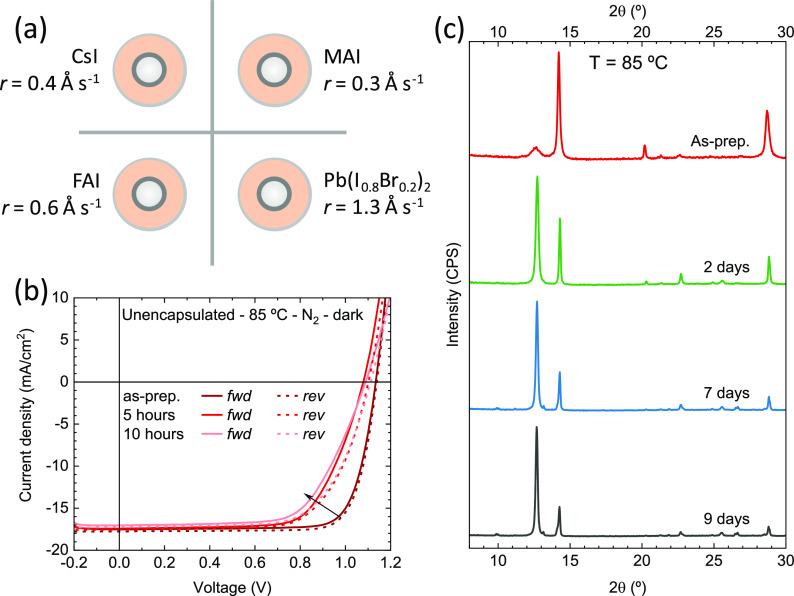
(a) Schematics of the deposition sources layout used in the vacuum processing of CsMAFA triple-cation perovskite films. Materials and corresponding deposition rates (*r*) are also reported. (b) *J*–*V* curves for a CsMAFA p-i-n solar cell taken at different times with the device kept at 85 °C on a hot plate in nitrogen atmosphere. The *J*–*V* curves are collected in forward (from short to open circuit, solid line) and reverse scan directions (from open to short circuit, dashed line). (c) XRD patterns for CsMAFA triple-cation perovskite films measured periodically ex situ during thermal stress.

The current–density versus voltage (*J*–*V*) curves under simulated solar illumination for a representative CsMAFA solar cell are reported in Figure 1b (statistics on the PV parameters are provided in Figure S4a). The solar cells showed a high fill factor (FF, 80% on average), indicating an efficient charge extraction of the photogenerated charge carriers. We also observed negligible hysteresis between the forward and reverse scans, which suggests that either ion migration or interface recombination (or both) are suppressed in these perovskite solar cells.^64,65^ The average short-circuit current density (*J*_sc_) and open-circuit voltage (*V*_oc_) were 17.4 mA cm^–2^ and 1157 mV, respectively, standing at 80% of the theoretical maximum as described by the radiative limit for a semiconductor with an *E*_g_ = 1.73 eV.^66^ The resulting average PCE was found to be 16.0%, with maximum values of 16.4%. We then evaluated the stability of the triple-cation CsMAFA perovskite devices via maximum power point (mpp) tracking under illumination (Figure S4c). The devices were encapsulated with a UV-curable resin and a glass slide, and the stability was evaluated in a nitrogen atmosphere at RT to minimize the effect of environmental factors. Under these operational conditions, the solar cell exhibited a limited stability, reaching 90% of the initial PCE after only 40 h of continuous operation. We further tested the properties of the devices upon storing them in inert atmosphere at 85 °C and periodically measured their *J*–*V* characteristics (Figure 1b). The CsFAMA solar cells were also not stable under thermal stress, as in only 5 h the *V*_oc_ and especially the FF were found to be strongly reduced (to approximately 1.1 V and 65%, respectively), a degradation which continued in the following time.

In order to shed light on the degradation mechanism leading to the fast loss of PCE of the CsMAFA triple-cation perovskite solar cells, we analyzed the XRD patterns of films under light and thermal stresses. In particular, different equivalent samples (from the same vacuum deposition run) were kept either under constant illumination (1 sun equivalent intensity) at 35 °C (Figure S5) or on a hot plate at 85 °C (Figure 1c) and periodically analyzed ex situ via XRD. Under light soaking (Figure S5) we observed a moderate rise in the PbI_2_ signal (2θ = 12.7°) during the first 2 days, which then remains stable, suggesting that light-soaking under AM 1.5G alone is not a factor of degradation. However, under constant thermal stress at 85 °C (Figure 1c), the degradation into crystalline PbI_2_ is accelerated, with its characteristic peak at 2θ = 12.7° becoming the most intense signal after only 2 days of thermal treatment. Other new nonperovskite phases are also observed. These are ascribed to δ-CsPbI_3_ and δ-FAPbI_3_ yellow phases (see Figure S6 for better visualization and peak assignment).^67^ These findings indicate that the inclusion of bromide might hinder the high thermal stability of vacuum-deposited perovskites, which was otherwise demonstrated for pure-iodide formulation.^63,69,72^

To stabilize the perovskite composition, we added GA^+^ as a fourth A-site cation. FAI can be sublimed with a stable rate of 0.6 Å s^–1^ at an approximate temperature of 155–160 °C, when pure GAI also sublimes (although with a slightly lower rate, *r* ≈ 0.2 Å s^–1^). As has been previously shown, only a small amount of GA^+^ is needed in order to structurally stabilize the perovskite without undermining device functioning.^57,60,61^ For this reason, we prepared a mixture of FAI:GAI with molar ratio 10:1 (see the Supporting Information for details) and sublimed it from the same crucible and at the same rate as for the triple-cation CsMAFA perovskites (schematics and summary in Figure 2a). The XRD characterization of the as-deposited quadruple-cation CsMAFAGA perovskite films is reported in Figure 2b. As with the triple-cation perovskite (Figure S2), we obtained a cubic perovskite with a similar lattice parameter of 6.22 Å. In principle, this suggests that guanidinium is not incorporated in large amounts within the crystal lattice (which would lead to a lattice expansion) in as-deposited films. Nevertheless, there are very marked effects on the material upon the addition of GA^+^, in particular the significant reduction of crystalline PbI_2_ and a much more pronounced crystalline orientation of the perovskite film, with respect to the GA^+^-free triple-cation perovskite films. The absorbance spectra of a 500 nm thick wide-bandgap CsMAFAGA perovskite film is reported in Figure 2c, showing again the expected perovskite absorption profile, with absorbance >1 for wavelength below approximately 550 nm and *E*_g_ = 1.72 eV, as estimated from the corresponding Tauc plot (Figure 2d). The photoluminescence spectrum (Figure 2e), obtained upon illumination with a 515 nm laser at carrier concentration equal to 1 sun illumination, shows a maximum at 1.727 eV and no halide segregation in as-prepared films.

**Figure 2:**
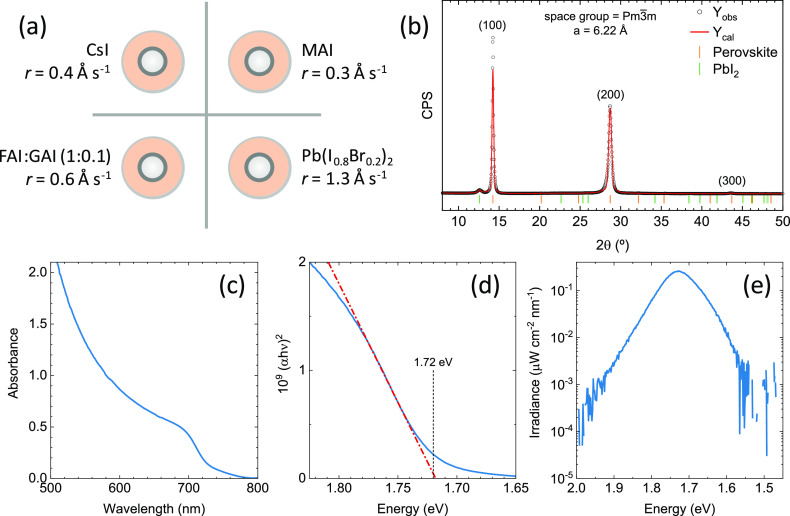
(a) Schematics of the deposition sources layout used in the vacuum processing of CsMAFAGA quadruple-cation perovskite films. Materials and corresponding deposition rates (*r*) are also reported. (b) XRD characterization of as-deposited triple-cation perovskite thin film on ITO. Observed (experimental) intensities are marked with open circles, Le Bail fit is represented in red, and Braggs’ reflection for the two different phases are indicated with vertical markers of different colors. Diffraction planes for the perovskite phase are indicated, as well as the considered space group and lattice parameter. (c) Optical absorption spectra of a 500 nm thick film with corresponding (d) Tauc plot to estimate the bandgap. (e) Calibrated absolute photoluminescence spectra of the same film upon excitation with a 515 nm laser light source.

In view of the low thermal stability of the triple-cation CsMAFA perovskite films, and in order to investigate the effect of the addition GA^+^, we initially assessed the stability of quadruple-cation CsMAFAGA perovskite films under light and thermal stresses. As described before, equivalent samples from the same vacuum deposition run were kept either under constant light soaking at 35 °C (Figure 3a) or on a hot plate at 85 °C (Figure 3b) and periodically analyzed ex situ via XRD. In both cases, the stability is remarkably improved with the addition of GA^+^. Note that the timespan in Figure 3 is 37 days, compared to the rapid degradation in only a few days observed for the triple-cation perovskites. The XRD of the CsMAFAGA perovskite films does not show a marked increase in PbI_2_ or other phases even after 5 weeks. Aside from this main observation, several additional transformations can be deduced from the XRD analysis. First, an apparent peak splitting occurs after 2 days at 85 °C or 28 days under light soaking. It could be thought that such a peak splitting indicates the coexistence of two phases. Nevertheless, the XRD signal can be fitted with a single lower-symmetry (orthorhombic) perovskite phase, isostructural to γ-CsPbI_3_ (see Figure S7).^73^ In any case, this phase transition (or phase segregation) appears to be a transitional stage, as the diffractogram after 1 week at 85 °C corresponds again to a single cubic perovskite phase. Interestingly, this new cubic perovskite phase (space group *Pm*3̅*m*) has a larger lattice parameter *a* = 6.23 Å, which can be qualitatively observed by the shift to lower diffraction angles of the main perovskite peaks. The reason for the cubic lattice expansion between the pristine sample and the annealed sample is not fully elucidated (all measurements are carried out at room temperature) but could be due to the partial incorporation of guanidinium in the crystal lattice triggered by thermal stress. Furthermore, we observe a clear peak sharpening between pristine and annealed samples. A detailed microstructural analysis (Figure S8) based on the whole-pattern Le Bail fit of the XRD signal and considering instrument resolution reveals that this peak sharpening is not strongly related to crystallite growth (note that crystallites are not identical to “grains” or domains observed by SEM)^74^ but rather to a release in microstrain. This, linked to the previous observation, suggests that the GA^+^ cations are not incorporated in the crystal lattice of the as-deposited films but rather are located at the grain boundaries. We deduce this because initially, the CsMAFAGA quadruple-cation perovskite has the same lattice constant as the CsMAFA triple-cation perovskite. This implies that the GA^+^ cations are acting as structural defects (e.g., at grain boundaries), which induces microstrain.

**Figure 3:**
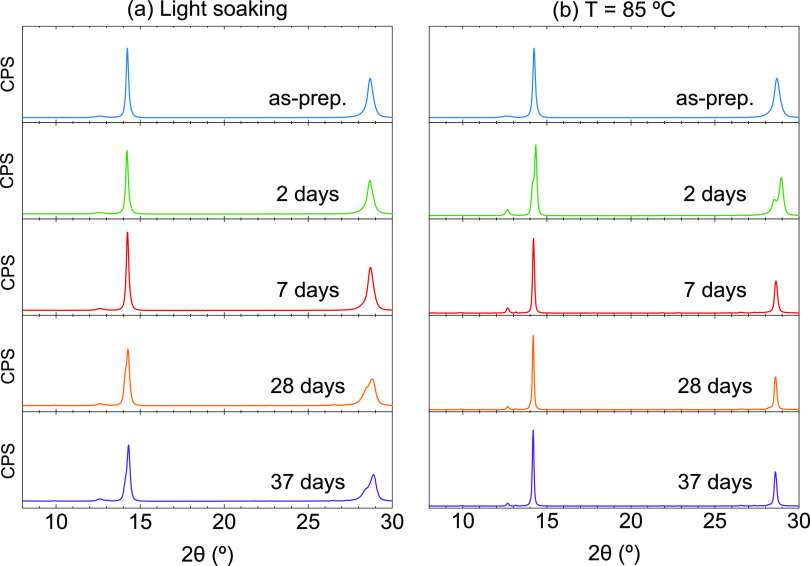
XRD patterns for quadruple-cation CsMAFAGA perovskite films measured periodically ex situ during (a) light soaking or (b) thermal stress.

As the films are thermally stressed, the GA^+^ cations might be incorporated to some extent within the crystal, leading first to a transition to a lower-symmetry perovskite phase and ultimately to the stabilization of a cubic phase with larger unit cell volume and released microstrain. It must be noted that the use of multication perovskites has already been shown to play an important role in such a strain release.^75,76^ Finally, we also observed a minor but persistent contribution from δ-CsPbI_3_ and δ-FAPbI_3_ after 1 week of thermal stress (see Figure S9 for better visualization). The presence of these yellow phases, which remains marginal even after 5 weeks, seems not to be detrimental for the perovskite film stability and could also partly contribute to its stabilization by suppressing ion migration, as recently demonstrated by others.^77^

The quadruple-cation CsMAFAGA wide-bandgap perovskite films were incorporated in thin-film solar cells with the same p-i-n configuration as described for the triple-cation perovskites in the previous section. The *J*–*V* curves (forward and reverse bias) under simulated solar illumination for a representative CsMAFAGA solar cell are reported in Figure 4a (statistics on the PV parameters are reported in Table S1). The solar cells showed a FF > 80%, indicating efficient charge extraction of the photogenerated charge carriers, similar to the above-described triple-cation perovskite solar cells. We again observed negligible hysteresis between the forward and reverse scans and measured *J*_sc_ and *V*_oc_ of 17.3 mA cm^–2^ and 1148 mV, respectively, essentially unaltered as compared to the triple-cation perovskite devices, indicating that the addition of GAI does not undermine (nor improve) the device functioning. The solar cells were encapsulated and the stability was initially evaluated in a nitrogen atmosphere at RT to minimize the effect of environmental factors on the degradation. The mpp tracking of the CsMAFAGA solar cells exhibited a remarkably enhanced stability, with the PCE being unaltered after 40 h of continuous operation.

**Figure 4:**
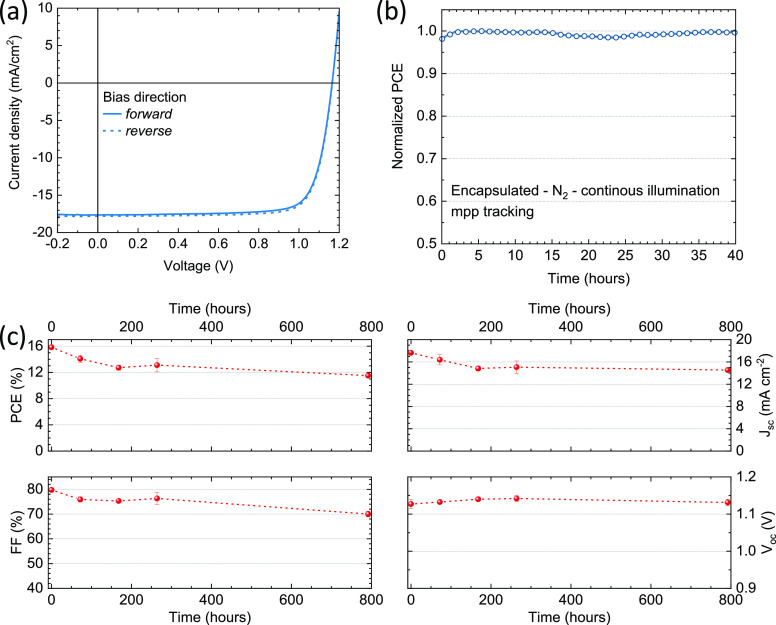
(a) Current density vs voltage (*J*–*V*) curves under illumination for a representative solar cell using a quadruple-cation CsMAFAGA perovskite as the absorber layer. The *J*–*V* curves are collected in forward (from short to open circuit, solid line) and reverse scan directions (from open to short circuit, dashed line). (b) Maximum power point tracking under simulated solar illumination for an encapsulated CsFAMAGA device, measured in inert atmosphere. (c) Shelf life thermal stability test for a similar solar cell: PV parameters extracted from *J*–*V* curves taken at different times for a device kept at 85 °C on a hot plate in nitrogen atmosphere.

We further tested the stability of the devices by storing them at open-circuit conditions in an inert N_2_ atmosphere at 85 °C and periodically measured their *J*–*V* characteristics under simulated 1 sun solar light at room temperature. Independent of the initial performance, we observed an increase in the FF of the solar cells (up to approximately 80%) and a small decrease of the *V*_oc_ (of about 20–25 mV). This does not change the initial efficiency of a well working device, but it results in a net performance improvement for faulty pixels, which are found to be working correctly after annealing at 85 °C for 1 h (Figure S10). The quadruple-cation CsMAFAGA solar cells were found to be very stable under thermal stress as compared to the triple-cation analogues (Figure 4c). The PCE dropped to about 80% of the initial value after 1 week of testing, and was found to be still above 75% of the initial PCE after 700 h (roughly 1 month) of continuous thermal stress at 85 °C, in analogy with the structural stability observed and describe in Figure 4. The PCE loss under thermal stress was mainly caused by small reductions in the *J*_sc_ and FF, while the *V*_oc_ was stable throughout the first month of the test (Figure 4c).

In summary, we present a strategy to increase the complexity of the formulation of vacuum-deposited lead halide perovskites films by multisource deposition and premixing both inorganic and organic components. We applied this method to the preparation of wide-bandgap CsMAFA triple-cation perovskite solar cells, which were found to be efficient but not stable, in particular when stressed at 85 °C. In an attempt to improve the stability, we added another A-site cation, guanidinium (GA^+^), to the perovskite formulation. The resulting CsMAFAGA quadruple-cation perovskite films showed much improved thermal stability, with no sign of material degradation (not even by XRD) even after more than a month at 85 °C. Microstructural analysis suggests that GA^+^ is initially not incorporated in the crystal structure, but it rather accumulates at the grain boundaries. However, during thermal stressing, a transition to a lower-symmetry perovskite phase and ultimately a stabilization of the cubic phase with larger unit cell volume is observed, indicating the incorporation of some GA^+^ into the crystal (see schematics in Figure S11). When used in solar cells, the wide-bandgap CsMAFAGA quadruple-cation perovskite showed similar performance but enhanced thermal stability (as compared to the triple-cation perovskite), comparable to what is observed for bromide-free vacuum-deposited perovskites. Future work will focus on a variety of different strategies, for example, halide alloying,^78^ the use different large ammonium cations,^79^ and/or the study of MA-free formulations,^80,81^ which have the potential to further enhance the thermal stability of the perovskite.

## Supporting Information Available

Experimental methods, SEM analysis, EDX, details of structural XRD analysis, optical absorption and luminescence data, device statistics and complementary data (PDF)
