# Supplementary material for: Quadruple-Cation Wide-Bandgap Perovskite Solar Cells with Enhanced Thermal Stability Enabled by Vacuum Deposition
Source: ACS Energy Lett. 2022 Mar 18;7(4):1355–63. doi: 10.1021/acsenergylett.2c00304 (PMC9004330; doi:10.1021/acsenergylett.2c00304)
Supplement: Supplementary file 1 — nz2c00304_si_001.pdf [file nz2c00304_si_001.pdf]

SUPPLEMENTARY INFORMATION

Quadruple-cation wide bandgap perovskite solar cells with enhanced thermal stability enabled by vacuum deposition

Isidora Susic,[\$] Lidón Gil-Escrig,[\$] Francisco Palazon, Michele Sessolo and Henk J. Bolink*

Instituto de Ciencia Molecular, Universidad de Valencia, C/ Catedrático J. Beltrán 2, Paterna 46980, Spain. E-mail: michele.sessolo@uv.es

[\$]I.S and L. G.-E. contributed equally to this work

Experimental Methods

Materials: Poly(triaryl)amine (PTAA), fullerene (C60) and $C(NH_2)_3I$ (GAI) were purchased from Merck KGaA. PbI_2, CH_3NH_3I (MAI) and bathocuproine (BCP) were purchased from Luminescence Technology Corp. $CH(NH_2)_2I$ (FAI) was purchased from Greatcell Solar. $PbBr_2$ was obtained from Alfa Aesar. All materials were used as received.

Thin films and solar cells fabrication: (ITO-coated) glass substrates were subsequently cleaned with soap, water and isopropanol in an ultrasonic bath, followed by 20 min UV-ozone treatment. All further processing was carried out in nitrogen-filled gloveboxes. PTAA solutions (1.5 mg mL^{-1}) were spin-coated on the substrates at 3000 rpm and annealed on a hot plate at 100 C for 10 min. The substrates were transferred to a vacuum chamber integrated in a nitrogen-filled glovebox and evacuated to a pressure of 10^{-6} mbar for the perovskite deposition. The chamber is equipped with four evaporation sources (Creaphys) and with independent temperature controllers and shutters. All sources have a dedicated quartz crystal microbalance (QCM) sensor above, and an additional one is installed close to the substrates for the overall deposition rate measurement. All sources were individually calibrated for their respective materials and no cross-reading between the different QCMs is ensured by the relative position of the sources, shutters, sensors. For thickness calibration, we individually sublimed each material, and a calibration factor was obtained by comparing the thickness inferred from the QCM sensors with that measured with a mechanical profilometer (Ambios XP1). During the perovskite deposition, the pressure of the chamber was maintained at $8 \cdot 10^{-6}$ mbar and the substrates were kept at room temperature. Typical sublimation temperatures for the precursors were approximately 155 ºC for FAI or FAI:GAI (r = 0.6 Å s^{-1}), 125 ºC for MAI (r = 0.3 Å s^{-1}), 310 ºC for $Pb(I_{1-x}Br_x)_3$ (r = 1.3 Å s^{-1}), and 485 ºC for CsI (r = 0.4 Å s^{-1}). The FAI:GAI mixture was prepared by weighting 1 g of FAI and 0.11 g of GAI in a vial, and by mixing them with a SpeedMixer^{TM} (DAC 150.1 FVZ) for 5 minutes at 3500 rpm. After mixing, they are transferred to the alumina crucible used for the sublimation. The mixed halide precursor $Pb(I_{0.8}Br_{0.2})_2$ was prepared by mixing in an alumina crucible 1 g of PbI_2 and 0.2 g of $PbBr_2$, and by heating them at 350 ºC for 5 minutes after complete melting of the mixture. Charge extraction layers were deposited in a second chamber integrated in a nitrogen-filled glovebox. The deposition rate for C_{60} was 0.5 Å s^{-1} while the thinner BCP

layer was sublimed at 0.2 Å s^{-1}. Ag was deposited in a third vacuum chamber from alumina-coated aluminum boats, and by applying currents ranging from 2.0 to 4.5 A.

Materials and device characterization: Absorption spectra were collected using fiber optics based Avantes Avaspec2048 Spectrometer. The photoluminescence spectra were measured with an Avantes Avaspec2048 spectrometer and films were illuminated with a diode laser of Integrated Optics, emitting at 515 nm. All the spectra were collected with an integration time of 1 s. The crystalline structure of the thin films was studied by X-ray diffraction (XRD). The patterns were collected in Bragg-Brentano geometry on an Empyrean PANalytical powder diffractometer with a copper anode operated at 45 kV and 40 mA. Further analysis including Le Bail fits were performed with Fullprof software. Microstructural analysis is performed based on these whole-pattern fits, considering instrumental resolution. In short, an instrument resolution file is provided based on previous measurement and analysis of a single crystal. Thus, instrumental broadening is taken into account for microstructural analysis. Whole-pattern Le Bail fits are then performed using a Thompson-Cox-Hastings pseudo-Voigt line shape. Parameters for the Gaussian and Lorentzian contributions of the pseudo-Voigt function are optimized by fitting of the experimental data. Importantly, these parameters are independent for every different phase present in the diffractograms so that the existence of multiple phases in a single diffractogram is not problematic. Obviously fitting of different diffractograms are also independent. Eventually, average crystallite size and strain values can be derived for every phase of every diffractogram. These characteristics can be represented in a Williamson-Hall plot for easy visualization. Note however that peak width parameters are not independently fitted for separate peaks within the same phase as the fitting procedure is a whole-pattern deconvolution. Thus, the Williamson-Hall plots present perfectly linear data.

Scanning Electron Microscopy (SEM) images were performed on a Hitachi S-4800 microscope operating at an accelerating voltage of 2 kV over platinum-metallized samples. The *J-V curves* for the solar cells were recorded using a Keithley 2612A SourceMeter in a -0.2 and 1.2 V voltage range, with 0.01 V steps and integrating the signal for 20 ms after a 10 ms delay, corresponding to a scan speed of about 0.3 Vs^{-1}. The devices were illuminated under a Wavelabs Sinus 70 LED solar simulator. The light intensity was calibrated before every measurement using a calibrated Si reference diode. Solar cell stability measurements were recorded using a maximum power point tracker

(mppt) system, with a white LED light source under 1 sun equivalent, developed by Candlelight. During the mppt measurements, a flow of N_2 gas was used and temperature was kept at 25 ºC using a water-circulating cooling system. Thermal stability was assessed by leaving the thin films or the encapsulated solar cells on a hot plate at 85 ºC in a nitrogen glove box, and by periodically characterized them ex situ.

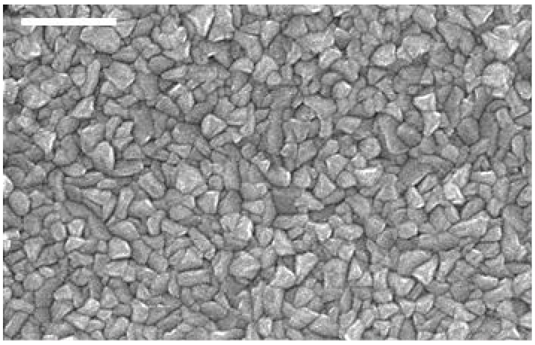

Figure S1. SEM picture of the surface of an as-prepared CsMAFA triple-cation perovskite. Scale bar is 500 nm.

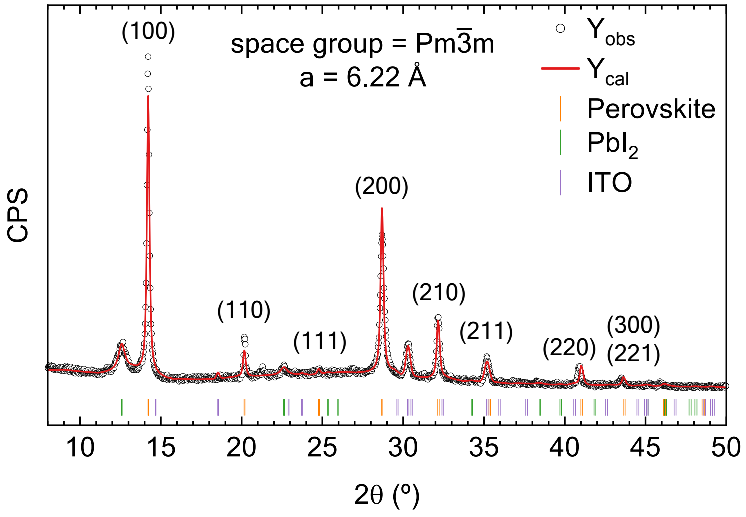

Figure S2. XRD characterization of as-deposited triple-cation perovskite thin film on ITO-coated glass slides. Observed (experimental) intensities are marked with open circles, Le Bail fit is represented in red and Braggs' reflection for the three different phases are indicated with vertical markers of different colors. Diffraction planes for the perovskite phase are indicated, as well as the considered space group and lattice parameter.

XRD analysis of triple cation perovskites: The cubic symmetry may be questioned, in view of existing stable tetragonal and orthorhombic perovskite phases. While the calculation of a tolerance factor in multi-cation and multi-anion formulations is not straightforward, we shall note that the relatively high amount of Br^- here (around 15-20% as evaluated by EDX, Figure S12) enhances the stability of Cs-containing perovskites.[1] Furthermore, the use of methylammonium in this triple-cation perovskite may enhance the stability of the cubic phase with regards to FA-Cs double cation perovskites as in the reference cited before.[2] Most importantly, no unassigned peaks and particularly no double peaks are seen around 2θ = 14° or 2θ = 28.5° which points to a cubic phase (see for example Figure 5 and S3 with the corresponding discussion, where this is not the case and the signal is fitted with an orthorhombic perovskite phase). Of course these arguments cannot completely rule out the assignment of the diffraction signal to a tetragonal phase with nearly identical lattice parameters or the coexistence of cubic and tetragonal phases, which would be very difficult to assess on highly-oriented thin films. Yet, considering the good fit obtained with a single cubic phase and the above discussion, we believe it is a reasonable assumption to consider a single cubic phase here.

References:

[1] R. E. Beal, N. Z. Hagström, J. Barrier, A. Gold-Parker, R. Prasanna, K. A. Bush, D. Passarello, L. T. Schelhas, K. Brüning, C. J. Tassone, H.-G. Steinrück, M. D. McGehee, M. F. Toney, A. F. Nogueira, *Matter* **2020**, *2*, 207.

[2] G. A. Tosado, Y.-Y. Lin, E. Zheng, Q. Yu, *J. Mater. Chem. A* **2018**, *6*, 17426.

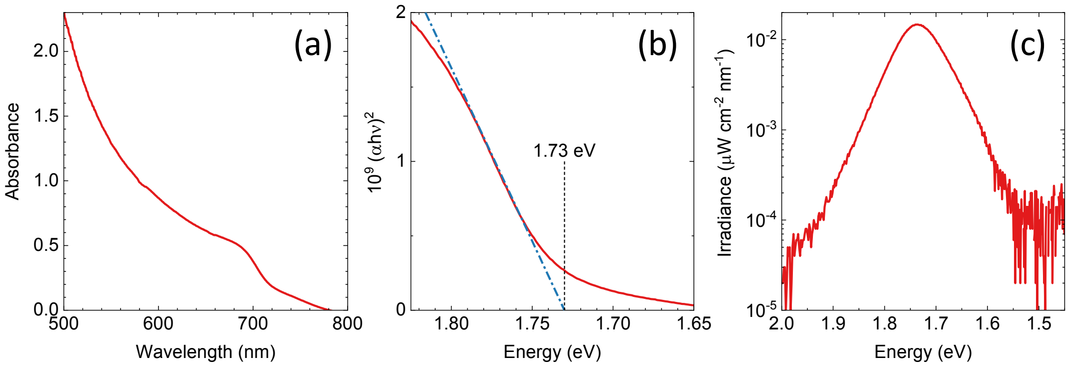

Figure S3. (a) Optical absorption spectra of a 500 nm thick film with corresponding (b) Tauc plot to estimate the bandgap. (c) Calibrated absolute photoluminescence spectra of the same film upon excitation with a 515 nm laser light source.

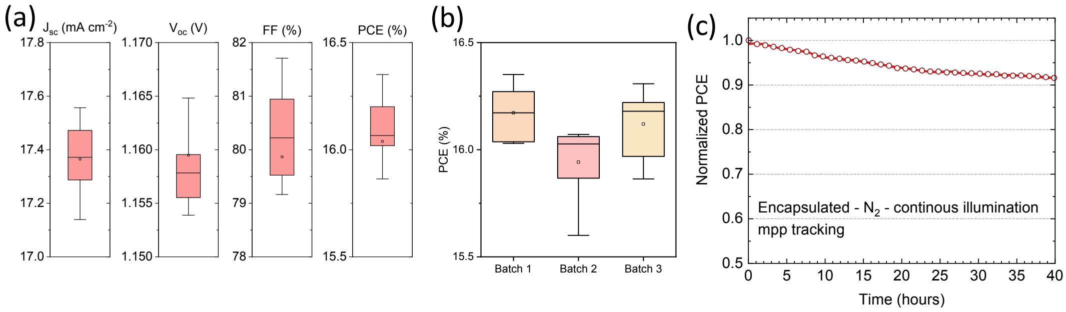

Figure S4. (a) Statistics of the photovoltaic parameters from 16 CsFAMA perovskite solar cells prepared in (b) 3 different batches. (c) Maximum power point tracking under simulated solar illumination for an encapsulated CsFAMA device, measured in inert atmosphere.

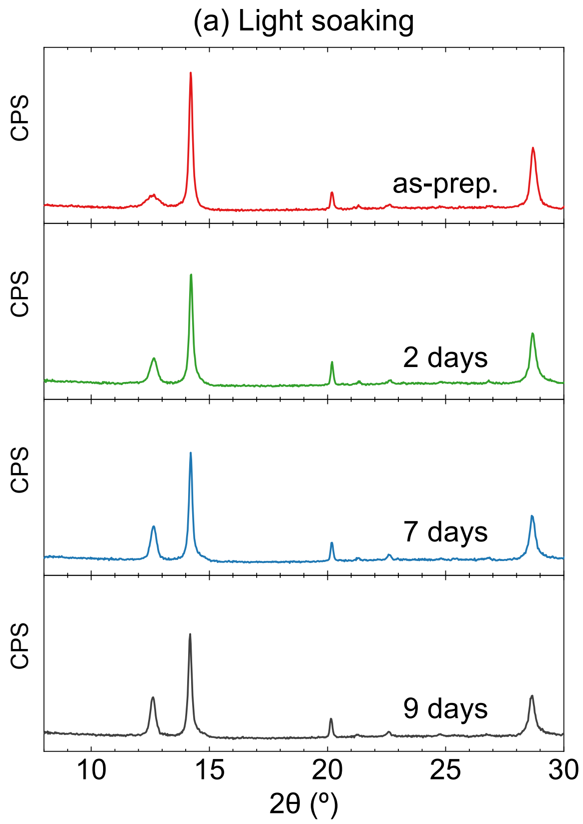

Figure S5. XRD patterns for CsMAFA triple-cation perovskite films measured periodically ex situ during light soaking at 1 sun equivalent intensity.

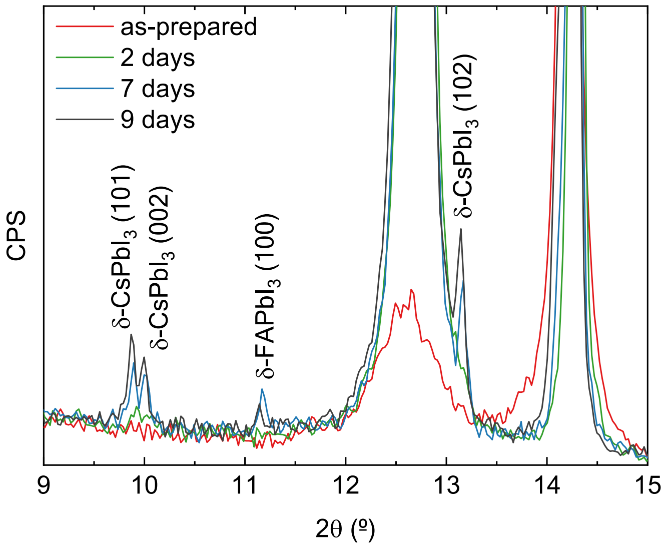

Figure S6. Low angle XRD patterns for CsMAFA triple-cation perovskite films upon ageing at 85 ºC, highlighting the formation of yellow phases.

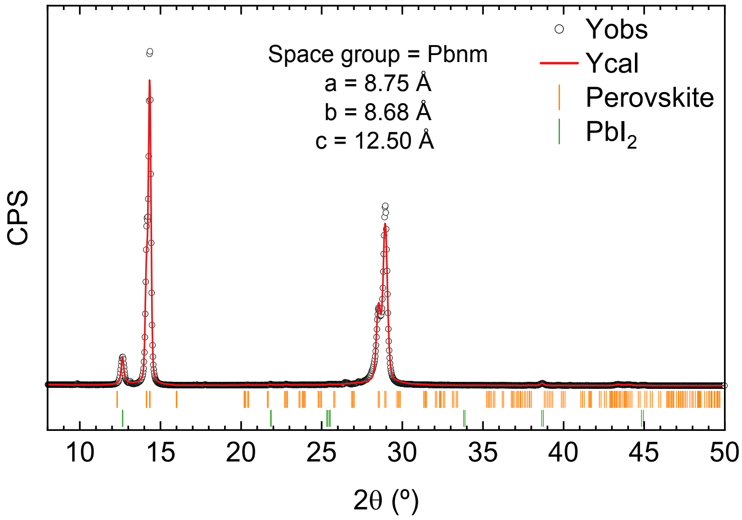

Figure S7. Whole-pattern fit of quadruple-cation CsMAFAGA perovskite thin film after 2 days at 85 ºC. A single orthorhombic perovskite phase is considered in addition to PbI_2.

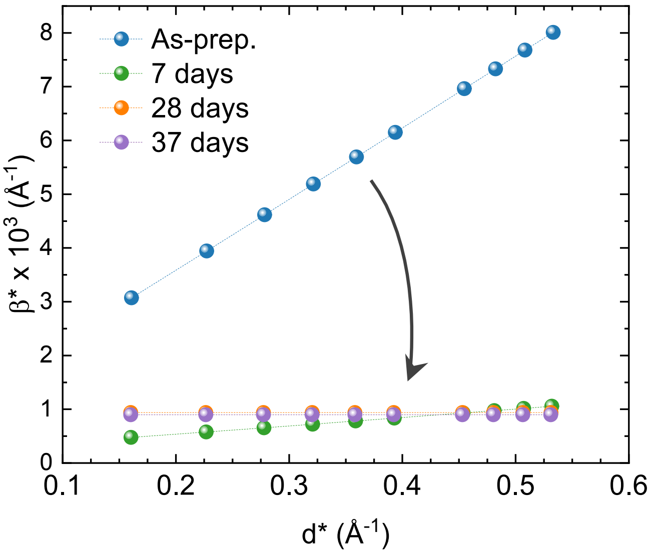

Figure S8. Williamson-Hall plots (inverse integral breadth versus inverse interatomic distance) for quadruple-cation films upon thermal stress. The most noticeable effect is a suppression of microstrain, related to the slope of the graph. Average crystallite size (inverse of extrapolated value at d* = 0) does not change significantly and remain around 100 nm.

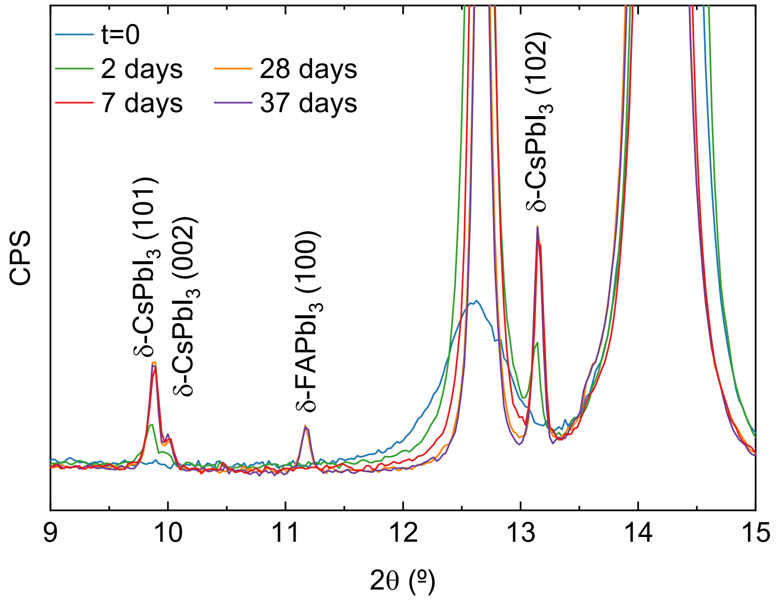

Figure S9. Low angle XRD patterns for quadruple-cation CsMAFAGA films upon ageing at 85 ºC, highlighting the formation of yellow phases.

Table S1. Average PV parameters extracted from the J-V characterization of quadruple-cation solar cells under simulated solar illumination.

Bias direction	V_{oc} *(V)*	J_{sc} *(mA cm^{-2})*	*FF (%)*	*PCE (%)*
Forward	1.148 ± 0.010	17.3 ± 0.2	82.4 ± 1.1	16.4 ± 0.2
Reverse	1.148 ± 0.070	17.3 ± 0.2	81.3 ± 1.5	16.1 ± 0.4

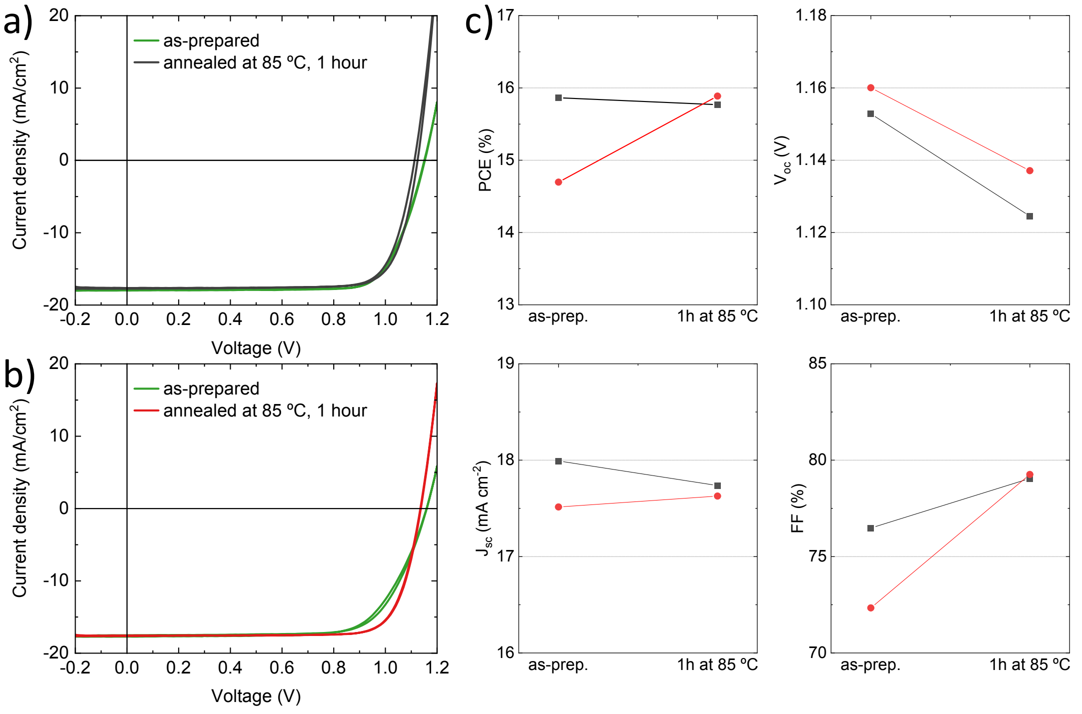

Figure S10. JV curves under illumination for quadruple cation CsMAFAGA perovskite solar cell, as-prepared and after annealing at 85 °C for 1 hour. The curves correspond in particular to (a) a well-working device and (b) for a faulty pixel. After annealing, the (c) PV parameters are essentially unvaried for the well working device, while they are found to be substantially improved for the initially faulty solar cells.

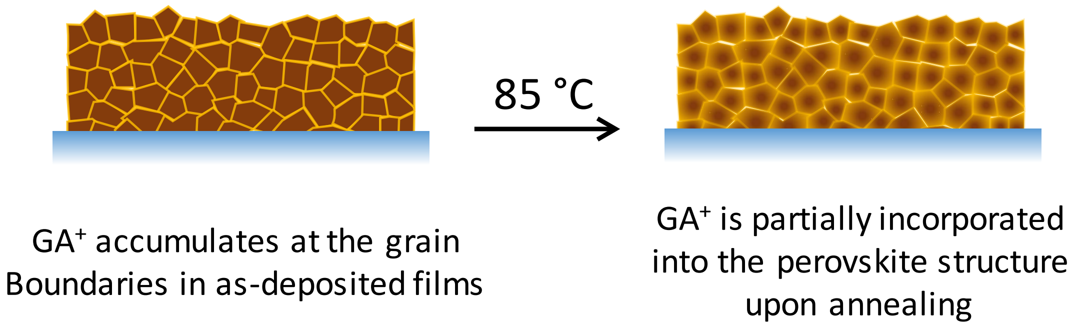

Figure S11. Schematics of GA+ dynamic incorporation in vacuum-deposited, quadruple cation CsMAFAGA perovskite films. GA^+ initially accumulates at the grain boundaries, but upon annealing is progressively incorporated into the perovskite structure, stabilizing the material and preventing degradation.

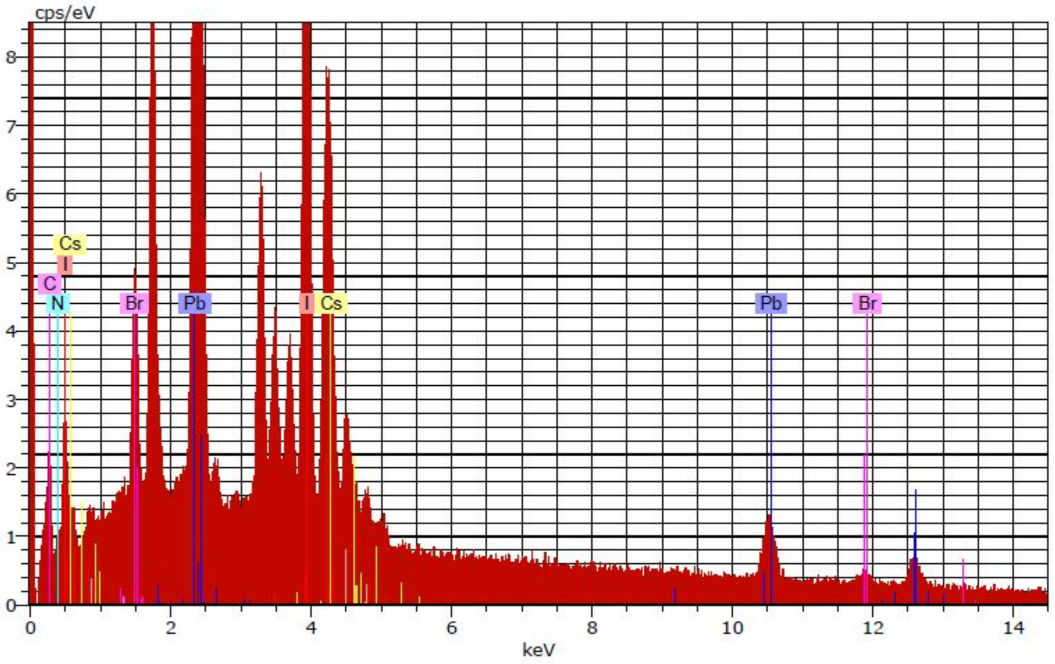

Spectrum: 1

Element	Series	Atom. C [at.%]	Error [wt.%]
Lead	L-series	11.86	0.9
Nitrogen	K-series	16.19	0.5
Carbon	K-series	34.90	0.6
Iodine	L-series	28.94	1.0
Bromine	K-series	4.60	0.2
Cesium	L-series	3.51	0.2
	Total:	100.00	

Figure S12. EDX analysis of an as-deposited wide bandgap perovskite film. From the atomic concentrations, we can derive a perovskite formula of the type $Cs_{0.3}A_xPb(I_{0.8}Br_{0.2})_3$, where A is the organic cation, whose content is not accessible due to the limitation of the technique and the exposure of the samples to atmosphere before measurements.
